# Spectroscopic and Theoretical Studies of Hg(II) Complexation with Some Dicysteinyl Tetrapeptides

**DOI:** 10.1155/2021/9911474

**Published:** 2021-07-23

**Authors:** Elliot Springfield, Alana Willis, John Merle, Johanna Mazlo, Maria Ngu-Schwemlein

**Affiliations:** ^1^Chemistry Department, Winston Salem State University, Winston-Salem, NC 27110, USA; ^2^Department of Chemistry and Biochemistry, University of North Carolina at Greensboro, Greensboro, NC 27402, USA

## Abstract

Tetrapeptides containing a Cys-Gly-Cys motif and a propensity to adopt a reverse-turn structure were synthesized to evaluate how *O*-, *N*-, *H*-, and aromatic *π* donor groups might contribute to mercury(II) complex formation. Tetrapeptides Xaa-Cys-Gly-Cys, where Xaa is glycine, glutamate, histidine, or tryptophan, were prepared and reacted with mercury(II) chloride. Their complexation with mercury(II) was studied by spectroscopic methods and computational modeling. UV-vis studies confirmed that mercury(II) binds to the cysteinyl thiolates as indicated by characteristic ligand-to-metal-charge-transfer transitions for bisthiolated S-Hg-S complexes, which correspond to 1 : 1 mercury-peptide complex formation. ESI-MS data also showed dominant 1 : 1 mercury-peptide adducts that are consistent with double deprotonations from the cysteinyl thiols to form thiolates. These complexes exhibited a strong positive circular dichroism band at 210 nm and a negative band at 193 nm, indicating that these peptides adopted a *β*-turn structure after binding mercury(II). Theoretical studies confirmed that optimized 1 : 1 mercury-peptide complexes adopt *β*-turns stabilized by intramolecular hydrogen bonds. These optimized structures also illustrate how specific *N*-terminal side-chain donor groups can assume intramolecular interactions and contribute to complex stability. Fluorescence quenching results provided supporting data that the indole donor group could interact with the coordinated mercury. The results from this study indicate that *N*-terminal side-chain residues containing carboxylate, imidazole, or indole groups can participate in stabilizing dithiolated mercury(II) complexes. These structural insights on peripheral mercury-peptide interactions provide additional understanding of the chemistry of mercury(II) with side-chain donor groups in peptides.

## 1. Introduction

Bis-cysteinyl sequences are present in many metalloproteins that play an important role in metal detoxification, regulation, homeostasis, or transport. The signature Cys-Xaa-Cys and Cys-Xaa-Xaa-Cys sequences are found in various types of metal-binding proteins such as metallothioneins [1, 2], MerP [3] and MerA [4], HAH1 [5], and Atox [6]. Coordination to cysteine residues dominates in these metal-protein associations. Accordingly, small peptide models containing such motifs have been designed to understand their biological role in some metalloenzymes, and in the rational design of peptide model systems as metal chelators. For example, an oligopeptide containing the Cys-Ala-Ala-Cys sequence, found in the MerP protein, retained its heavy metal-binding activity [7]. Constrained tetrapeptides, such as Cys-dPro-Pro-Cys, have also been designed to preorganize the two cysteine residues so that their thiol donor groups are positioned as “anchors” for mercury(II) coordination [8].

Although the arrangement or preorganization of metal coordinating groups/atoms is fundamentally important at the primary metal coordination site of metalloproteins, their stability or function is often also linked to their local environment. Such features have been demonstrated in the design principles of model systems for the binding and activation of dioxygen by transition-metal complexes. For example, Borovik and coworkers designed various tripodal ligands containing local intramolecular H-bonding networks around transition metal ions [9–11].

The modular nature of peptides makes them versatile in offering various possible types of noncovalent intramolecular local interactions, which can enhance the stability of metal-peptide associations. Therefore, molecular designs of bis-cysteinyl peptides containing side-chain groups that can stabilize the local environment or the metal coordination site may present advantageous cooperative binding effects. Bis-cysteinyl tetrapeptides with a sequence type Xaa-Cys-Gly-Cys have an intrinsic tendency toward adopting a type II *β*-turn. In this type of peptide reverse turn, it usually has glycine as the third residue from the amino terminal of the tetrapeptide backbone turn structure; and the 180° turn involves hydrogen bonding between the carbonyl oxygen of the first residue with the amino group hydrogen of the fourth residue [12]. Therefore, tetrapeptide Xaa-Cys-Gly-Cys could spatially position the cysteinyl thiolate groups on the same side of the peptidic turn structure to bind “soft” metal ions such as mercury(II). Its *N*-terminal residue presents the opportunity to position potential donor groups such as the indole side-chain of tryptophan, which is both electron-rich and possesses an *H*-donor [13]. It is well documented that this aromatic *π*-group can participate in various types of cation-*π* interactions or shield the coordinated metal against ligand exchange or oxidation [14–20]. In addition, the nonprotonated imidazole *N*_im_ in histidine is a strong metal coordinating ligand and is well known in the chelation of zinc in the “zinc-fingers” [21]. Kinetically stable mercury(II) complexes could be useful for various applications, including effective treatment of wastewater, regeneration of mercury adsorbents, and mercury chelation therapy. An understanding of how mercury ions form complexes with cysteinyl peptides, which contain auxiliary donor group(s), could provide useful insights for optimal mercury immobilization.

**Figure 1:**
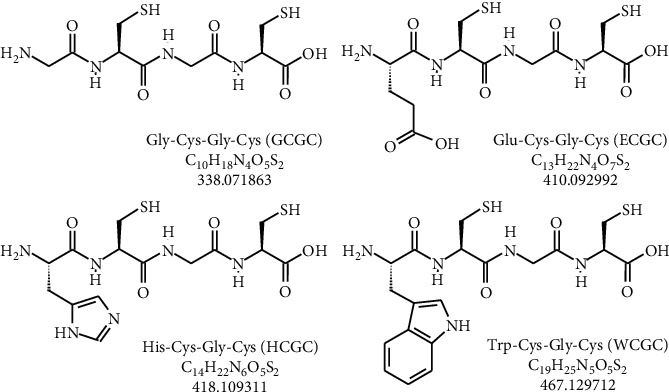
Chemical structures of bis-cysteinyl tetrapeptides and their monoisotopic masses.

Previously, we reported how the glutamyl carboxylate group in a tripeptide ligand, Cys-Glu-Cys, and the tryptophan indole group of a pentapeptide, Gly-Cys-Trp-Cys-Gly, affect mercury complex formation and their relative stabilities [22, 23]. In continuation of this work, the specific aim of this study is to gain an understanding of how proximal *N*-terminal amino acid side-chains, consisting of an *O*-, *N*-, *H*-, or *π*-donor, may contribute to complex stability in tetrapeptides containing a Cys-Gly-Cys reverse-turn motif. Accordingly, tetrapeptides Xaa-Cys-Gly-Cys, where Xaa is glycine (control peptide), glutamate, histidine, or tryptophan (Figure 1), were prepared and reacted with mercury(II) chloride. The mercury(II) coordination properties and peptide backbone-folding tendencies following interactions with mercury(II) were studied by spectroscopic methods. Electrospray ionization mass spectrometry (ESI-MS) was used to characterize the stoichiometry of complexes formed in ionized states. Optimized structures of each dithiolate-mercury(II) peptide complex were compared by molecular modeling. The proximity of the indole group to the mercury(II) coordination site was also investigated by fluorescence spectroscopy. The results of this study will provide some insights into how donor group(s) at the *N*-terminal residue may participate in mercury(II) complexation or overall complex stabilization.

## 2. Materials and Methods

### 2.1. Materials

Fmoc-Cys (Trt) (Wang Resin LL)-resin (where Fmoc is fluorenylmethyloxycarbonyl, and Trt is triphenylmethyl), 1-hydroxy-7-azabenzotriazole (HOAT), *O*-(7-azabenzotriazol-1-yl)-*N*, *N*, *N*′, *N*′-tetramethyluronium hexafluorophosphate (HATU), and Fmoc-amino acids were purchased from Novabiochem (San Diego, CA, USA). Ethyl cyano(hydroxyimino)acetate (Oxyma Pure) and Fmoc-His (Boc) were purchased from CEM, Corporation (Matthews, NC, USA). Collidine (2, 4, 6-trimethylpyridine, TMP), triisopropylsilane (TIS), 3, 6-dioxa-1, 8-octanedithiol (DODT), trifluoroacetic acid (TFA), diisopropylcarbodiimide (DIPCDI), dichloromethane (DCM), and mercury(II) chloride were obtained from Sigma-Aldrich (St. Louis, MO, USA). Mercury compounds are hazardous and should be disposed in designated chemical waste containers. HPLC grade water and acetonitrile, anhydrous ether, and N, N-dimethylformamide (DMF) were purchased from Fisher Scientific (Pittsburgh, PA, USA). Analytical and semiprep reversed-phase C-18 HPLC columns (Gemini: 300 Å, 5 *µ*m) were purchased from Phenomenex, Inc. (Torrance, CA, USA).

### 2.2. Preparation of Tetrapeptides

Tetrapeptides (Figure 1) were prepared by microwave-assisted solid phase peptide synthesis following the standard Fmoc-strategy by using a CEM Discovery microwave peptide synthesizer (CEM, Corp., Matthews, NC, USA) as previously described by our group for similar peptides [22, 24]. The analytical HPLC condition was 0% B to 30% B over 30 min with a flow rate of 1 mL/min, where solvent A is HPLC H_2_O with 0.1% TFA and solvent B is HPLC acetonitrile with 0.08% TFA. The retention times for these tetrapeptides are 9.7 min, 10.9 min, 9.6 min, and 21.9 min, respectively. Following purification by semipreparative HPLC, these peptides were obtained at >95% purity at an overall yield of 60%, 58%, 59%, and 43%, respectively. The purified peptides GCGC (molecular mass is 338.07 g/mol), ECGC (410.09 g/mol), HCGC (418.11 g/mol), and WCGC (467.13 g/mol) were characterized by ESI mass spectrometry. The observed mass for their [M+H]^+^ ion corresponds to the calculated values of 339.08, 411.10, 419.11, and 468.14, respectively (Figure S1).

### 2.3. UV-Vis Spectrophotometry

Stock peptide solutions were prepared in HPLC grade water previously degassed and purged with argon. The peptide concentration was determined by UV absorbance at 214 or 280 nm [25]. Each tetrapeptide solution was titrated with HgCl_2_ in 50 mM sodium phosphate at pH 7. The UV absorption spectra of titrated reaction mixtures were acquired on a dual beam Shimadzu UV-2401PC series UV-vis spectrophotometer. They were measured at room temperature in a 1 cm path-length quartz cuvette. A 2.5 mL solution consisting of 50 or 100 *µ*M peptide was titrated with aliquots of a 50 mM HgCl_2_ stock solution prepared in the same buffer. The reaction mixture was stirred for 1 min followed by an additional 2 min equilibration. The formation of the ligand metal charge-transfer (LCMT) band for the binding of Hg^2+^ to the thiolates was measured at 200 nm. The pH of each final reaction mixture was measured and was within 0.05 pH units of 6.6.

### 2.4. LTQ Orbitrap Mass Spectrometry

Samples were analyzed on an Orbitrap instrument, the Thermo Fisher Scientific LTQ Orbitrap XL mass spectrometer (Thermo Fisher, San Jose, CA), as described in our previous study [22]. Complexes of the peptides with Hg(II) were evaluated by reacting samples of Hg(II) and peptide solutions at a molar ratio of 1 : 1 in 5 mM ammonium formate, pH 7. The concentration of Hg(II) and the tetrapeptide were fixed at 7.5 × 10^−5^ M. The analysis was conducted after Hg(II) and peptide were reacted for 45 min. MS scans were acquired over an *m/z* range of 125–2000. Each sample injection acquired 200 scans. ESI-MS spectra are displayed by setting the base peak at 100% relative abundance, and by labeling the *m/z* value of the most intense peak in each isotopic cluster.

### 2.5. Circular Dichroism

CD spectroscopic measurements were conducted on a Jasco J-815 Circular Dichroism Spectrometer (Easton, MD, USA) equipped with a Peltier temperature-controlled cell holder (PTC-423 S/C) as previously reported [23]. Spectra were recorded at 25°C using a 0.1 cm path-length quartz cell with the following parameters: standard sensitivity = 100 mdeg; continuous scanning mode at 50 nm/min; bandwidth = 1.0 nm; response = 4 sec; data pitch = 0.1 nm. Peptide solutions were prepared at 50 *µ*M in 50 mM sodium phosphate at pH 7.

### 2.6. Fluorescence

Fluorescence quenching measurements were carried out on a FluoroMax spectrofluorometer (Horiba Scientific, NJ, USA), equipped with a Peltier temperature-controlled cell holder (SGL-POS QNW W/CIR). Tryptophan emission fluorescence was measured in the presence of increasing concentration of HgCl_2_. The fluorescence spectra were recorded using 10 *µ*M peptide solutions in a 3 mL quartz cell that has a path length of 1 cm. Mercury(II) chloride stock solution was prepared at 5 mM and added to the peptide in 50 mM sodium phosphate, pH 7. The excitation wavelength was set at 280 nm and emission spectra were recorded between 290 and 500 nm. The excitation and emission slit widths were fixed at 3 nm. The rate of scanning was set at 1 nm/s. Variable temperature Stern-Volmer plots were conducted at 25°C, 35°C, and 45°C.

### 2.7. Computational Modeling

The conformation space for each Hg(II)-peptide complex was explored using the Systematic pseudo-Monte Carlo Multiple Minimum (SPMC) search method implemented in Macromodel 11.9 [26] with the OPLS2005 force-field. The OPLS2005 molecular mechanics force-field has no parameters for mercury(II). Parameters for strontium (II) are available for the force-field and were tested as a substitute for mercury(II) since the charges are the same and sizes are similar. It was found that structural changes between force-field generated structures with strontium (II) and the final structures with mercury(II) were minor. Therefore, for conformation searching, strontium (II) was used in place of mercury(II) and then replaced with mercury in subsequent steps. For each complex, 100,000 conformations were generated and tested for similarity. Of the unique structures, those within 100 kJ/mol of the lowest OPLS2005 energy were further evaluated via M06-2X/LANL2DZ single-point energy calculations under vacuum conditions. Fifty structures with the lowest M06-2X/LANL2DZ energy advanced to the geometry optimization stage and high-level energy evaluation as described in the following paragraph.

Hg(II)-peptide complex geometries were optimized using the M06-2X density functional theory method combined with the 6-31G (d, p) basis set for polypeptide atoms and the Stuttgart/Dresden (SDD) basis set [27] with pseudopotential for 60 core electrons for the mercury atom. Others and we have applied the M06-2X method and obtained quality geometries for peptides and Hg(II)-peptide complexes [22, 28, 29]. As in our previous studies, single-point energies calculations were obtained using MP2 (full) with a 6-311G (d, p) basis set on peptide atoms and cc-pVDZ-PP basis set on mercury atom [22]. The IEFPCM (SMD) solvation model is used to model an aqueous environment in all calculations with default parameter except a van der Waals sphere size of 2.34 Å is used for the mercury atom. Vibrational frequencies, using the geometry optimization level of theory, were determined to ensure all optimized complexes were minima having no imaginary vibrational frequencies.

Structure stability was estimated using the Gibbs energy in solution (*G*_soln_) calculated by using (1), as previously described [23]. The Gaussian09 software was used for all M06-2X and MP2 calculations [30].

(1) $$\begin{array}{c} G_{\text{soln}}=G_{\text{gas}}+\Delta G_{\text{solv}}. \end{array}$$

## 3. Results and Discussion

### 3.1. Design and Synthesis of Bis-Cysteinyl Tetrapeptides

The bis-cysteinyl tetrapeptides (Figure 1) were prepared by microwave-irradiated solid phase peptide synthesis following the standard Fmoc-strategy as previously described for analogous compounds [22, 24]. Their sequence was designed to have a propensity to adopt the common *β*-turn involving four amino acids [12]. Glutamic acid, histidine, or tryptophan, consisting of *O*-, *N*-, *H*- or *π*-donor(s), respectively, was selected as the *N*-terminal amino acid. Its glycine analogue provides the reference compound to evaluate the how such donor groups may participate in peptide turn structure and complex formation. Crude peptides were purified to at least 95% purity by semipreparative high performance liquid chromatography (HPLC) and ESI-MS analysis confirmed the expected molecular mass.

### 3.2. UV-Vis Spectrophotometry

**Figure 2:**
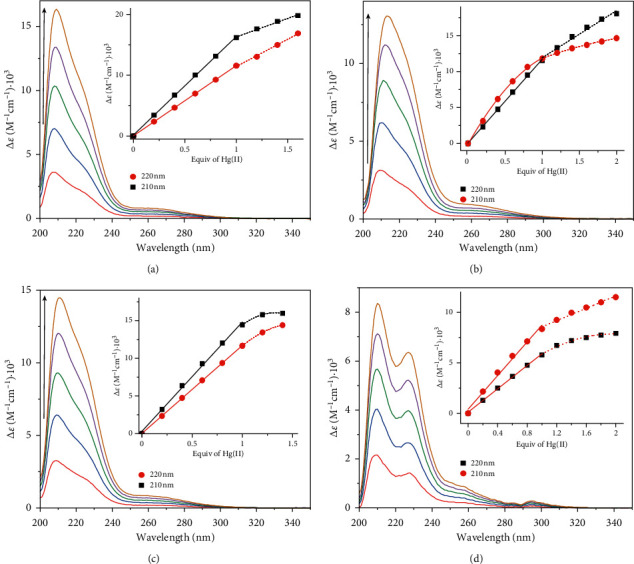
UV absorption difference spectra [Δ*ε* = *ε* (HgPeptide)-*ε* (Peptide)] correspond to **GCGC** (a), **ECGC** (b), **HCGC** (c), and **WCGC** (d) from titration with 0.2 to 1.0 mole equiv of HgCl_2_. Inset shows changes in extinction coefficient values at specified wavelengths versus mole equiv of HgCl_2_ added.

The formation of complexes between mercury(II) and each tetrapeptide was studied by monitoring the ligand-to-metal charge-transfer (LMCT) transitions for S---Hg bonds in the mid-UV energy range. The LMCT band was measured by subtracting the background spectrum of the peptide in the absence of mercury(II).  Figure 2(a) shows the absorption difference spectra for **GCGC** following titrations with increasing mole equivalence of mercury(II). A steady increase in the absorption bands centered at ca. 210 and 220 nm up to one equivalence of mercury(II) indicates the formation of a complex with 1 : 1 mercury to peptide stoichiometry. Changes in the 210 nm absorption difference band reflect changes in the peptide amide absorption (*n* to *π*^*∗*^) following structural changes in the peptide backbone when **GCGC** binds mercury(II). The ca. 220 nm absorption shoulder band exhibits an extinction coefficient of 11560 M^−1^ cm^−1^ at 1 : 1 mercury(II) to **GCGC** stoichiometry (Figure 2(a) inset). This is in agreement with values previously reported for linear, bisthiolated mercury complexes, which exhibit a characteristic high-energy LMCT band at ca. 220 nm [8, 23, 31]. In excess of mercury(II), the rate of increase in extinction coefficient at 210 and 220 nm changed. These new transitions may indicate the formation of polymetallic mercury species as noted in our previous work and by others [8, 23].

Similar trends were observed for the binding of mercury(II) to **ECGC** and **HCGC**. They exhibited an absorption band at ca. 220 nm with an extinction coefficient of 11560 and 11660 M^−1^ cm^−1^, respectively (Figures 2(b) and 2(c)). In contrast, the extinction coefficient at 220 nm for **WCGC** was significantly lower (5780 M^−1^ cm^−1^) (Figure 2(d)). Following titrations with increasing mercury(II), a new absorbance maximum band developed at ∼227 nm. As established previously by others, the *B*_*b*_ absorption band of the tryptophan indole ring around 220 nm will weaken and undergo a red shift when its *π* electrons interact with a cation [17, 32, 33]. For example, Okada and Miura reported such changes in the absorption spectra of Ctr4NT, a model peptide of Ctr4, a copper transport protein of fission yeast, when it binds Cu(I). They noted that the Cu(I)-bound peptide spectrum minus the free peptide spectrum showed a pair of negative/positive peak at 220/232 nm, a diagnostic red shift of the *B*_*b*_ band that indicates indole *π*-cation interaction [17]. In the absorption difference spectrum of **WCGC** (Figure 2(d)), a pair of minimum and maximum absorption bands at 220/227 nm are consistent with this red shift, which suggests that its indole group could be participating in some type of cation-*π* interaction. We have previously reported similar spectral changes for model pentapeptides whereby its tryptophan indole group could interact with mercury(II) or ammonium cations [23].

### 3.3. Electrospray Ionization Mass Spectrometry

**Figure 3:**
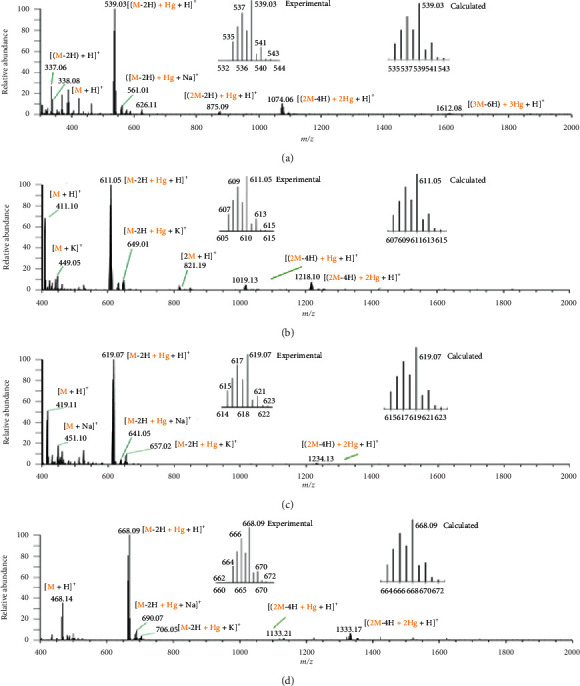
Electrospray ionization mass spectra of reaction mixtures containing 1 to 1 ratio of Hg(II):tetrapeptides. (a) **GCGC** + Hg^2+^, (b) **ECGC** + Hg^2+^, (c) **HCGC** + Hg^2+^, (d) **WCGC** + Hg^2+^, and the *m/z* value of the most intense peak in each isotopic cluster. Insets show the experimental and calculated mercury isotopic patterns for the 1 : 1 mercuriated peptide adducts.

Formation of the mercury-peptide complexes was also verified by ESI-MS. Reaction mixtures containing equimolar mercury(II) to peptide ratio were analyzed.  Figure 3 shows the source spectra for the tetrapeptides and their mercuriated adducts. The major mercury(II) complex detected for the four tetrapeptides corresponds to 1 : 1 mercury-peptide complex. Figures 3(a)–3(d) insets show that the experimental mercury isotopic pattern for the major 1 : 1 mercury-peptide adducts matches well with the calculated isotopic pattern [34]. They exhibit the signature isotopic pattern, which is consistent with the seven naturally occurring isotopes of mercury and their natural abundances. The *m/z* values of each 1 : 1 mercury-peptide adduct corresponds to double deprotonations, likely from two thiol groups to form thiolates, which then act as the coordinating donor groups to form the respective dithiolated mercury complexes. These mercury-peptide complexes also form cationized adducts with Na^+^ and/or K^+^. Minor mercuriated peptide dimers [1 : 2 Hg(peptide)_2_ and 2 : 2 Hg_2_(peptide)_2_] and trimer [3 : 3 Hg_3_(peptide)_3_] are also detected as cationized adducts. Their *m/z* values also correspond to double deprotonations from the peptide for each mercury associated with the respective complex.

### 3.4. Circular Dichroism

**Figure 4:**
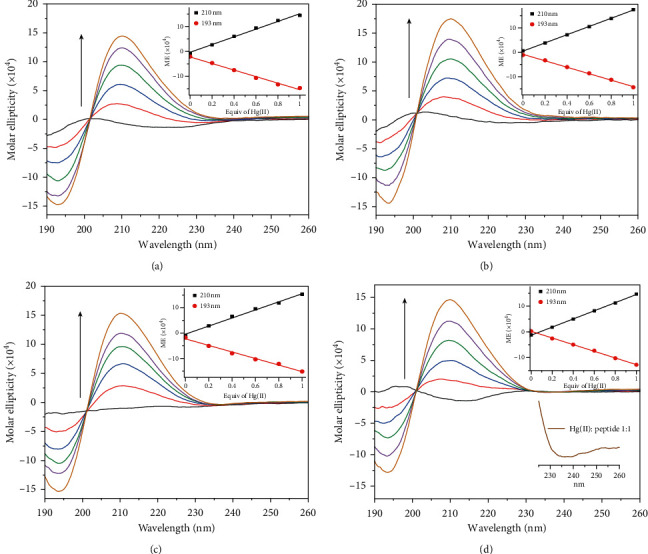
Circular dichroism spectra of 100 *μ*M tetrapeptides, (a) **CGCG**, (b) **ECGC**, (c) **HCGC**, and (d) **WCGC** following titrations with increasing mole equiv of HgCl_2_ as indicated by direction of arrow for 210 nm band. Insets show changes in molar ellipticity value at 193 nm and 210 nm versus mole equiv of HgCl_2_ added.

Figures 4(a)–4(d) show the CD spectra of each tetrapeptide before and after titrations with mercury(II) at 0.2 mol equiv increments. The free peptides **GCGC** (Figure 4(a)) and **ECGC** (Figure 4(b)) show a weak *nπ*^*∗*^-type transition negative band at ca. 222 nm, a weak positive band at ca. 200 nm, and a negative band at ca. 190 nm (*ππ*^*∗*^ transitions).  Figure 4(c) shows the CD spectrum of **HCGC**, which exhibits two low amplitude negative bands at 230 nm and 192 nm. The relatively more hydrophobic peptide, **WCGC**, shows two positive bands at ca. 225 nm and 197 nm, and a negative band at ca. 214 nm (Figure 4(d)). These relatively low intensity CD bands (less than ± 2 × 10^4^ deg M^−1^ cm^−1^) indicate that these free tetrapeptides exhibit very little secondary structure and do not form any dominant preorganized turn conformations. However, in the presence of increasing concentrations of mercury(II), their CD spectra intensified with the development of a positive band at 210 nm and a negative band at 193 nm. These CD spectra resemble a peptide backbone reverse turn corresponding to a *β*-turn type, reminiscent of a class B CD spectrum [35]. Theoretical calculations by Woody and others predicted that this turn type are characterized by a strong positive band between 200 and 210 nm (*nπ*^*∗*^ transition), and a strong negative band between 180 and 190 nm (*ππ*^*∗*^ transitions) [35]. As shown in Figures 4(a)–4(d), each peptide-mercury complex exhibits strong CD signals corresponding to this *β*-turn type. This reverse turn of the peptide chain could position the *N*-terminal side-chain group in proximity to the mercury(II) coordination site to participate in complex stabilization.

Figure 4 insets show an increase in the positive ellipticity at 210 nm and negative ellipticity at 193 nm that are linearly dependent on the concentration of mercury(II) in the solution. These spectra also exhibit one isodichroic point at ca. 200 nm indicating transitions to a common secondary structure following titrations with mercury(II) up to one-to-one mole equiv ratio. However, in excess of mercury(II), the intensity of the CD bands weakens. As observed in their corresponding UV spectra (Section 3.2), this may be due to the formation of polymetallic species, resulting in the loss of structural integrity.

In the presence of equimolar mercury(II), **WCGC** also showed a weak negative band ca. 235 nm (Figure 4(d) bottom inset showing magnified view). Yorita et al. earlier reported a similar development of a negative CD band at 223 nm, which was characterized as a signature CD band for a Cu(II)-Trp cation-*π* interaction [36]. Previously, we also reported a similar observation for a Hg(II)-Trp interaction [23]. Based on these comparisons and UV spectral changes of the indole *B*_*b*_ transition band for **WCGC** (Section 3.2), the indole group in **WCGC** could be undertaking a cation-*π* interaction, though it may be a weak or transient interaction based on the relatively small negative CD signal.

### 3.5. Tryptophan Fluorescence Spectroscopy

**Figure 5:**
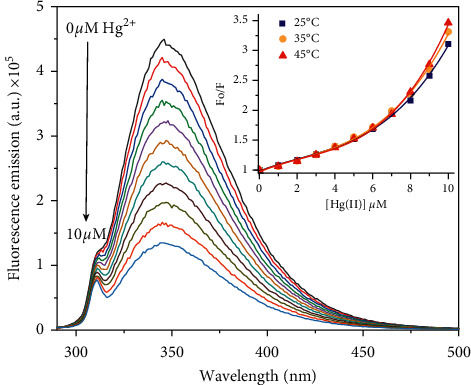
Fluorescence spectra of 10 *µ*M **WCGC** following titration with increasing concentrations of mercury(II) at 35°C. Inset: Stern-Volmer plots at 25°C, 35°C, and 45°C.

Figure 5 shows the fluorescence emission spectra of **WCGC** following titrations with mercury(II). About 70% of its intrinsic fluorescence is quenched after an equiv amount of mercury(II) was added. The fluorescence of the indole group in tryptophan is very sensitive to changes in polarity, and noncovalent interactions such as cation-*π* associations involving metal or ammonium ions [18, 37–40]. Its fluorescence emission can be quenched by mercury(II) via complex formation resulting in static quenching, or diffusive encounters that result in dynamic quenching [41]. Dynamic quenching is a diffusion-controlled process, which increases at higher temperature due to faster diffusion. In contrast, higher temperature usually decreases static quenching due to dissociation of weakly bound complexes [42]. Therefore variable temperature fluorescence quenching of **WCGC** by mercury(II) could be used to gauge the strength of any mercury(II)-indole-*π* interactions. Figure 5 (inset) presents the Stern-Volmer plot for the fluorescence quenching of **WCGC** by mercury(II) at 25°C, 35°C, and 45°C. These plots show an upward curvature, which is characteristic of fluorescence quenching via both complex formation and diffusive collisions. However, the small degree of temperature dependent quenching indicates that the tryptophan indole could participate in cation-*π* interaction with the coordinated mercury. This interaction could stabilize the mercury-peptide complex and possibly provide a hydrophobic shielding effect for the dithiolated mercury.

### 3.6. Computational Studies

**Figure 6:**
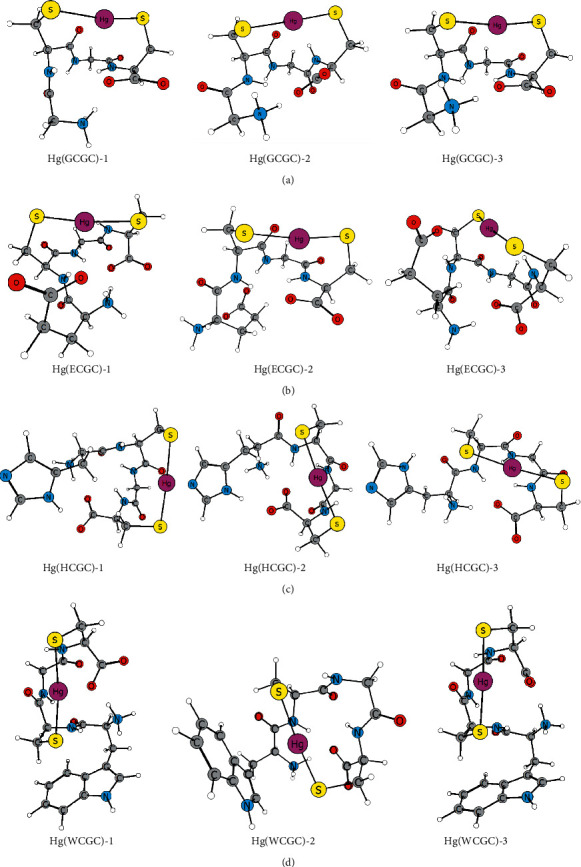
Three most stable Hg(XCGC) conformations (*G*_solution_) for each tetrapeptide where X is glycine (a), glutamate (b), histidine (c), and tryptophan (d). Complex with 1 designation is the most stable conformation in the group.

To understand how the *N*-terminal side-chain group may contribute to the structural stability of a XCGC peptide-mercury(II) complex in aqueous solution, 1 : 1 mercury(II)-peptide complexes were optimized and evaluated for stability.  Figure 6 shows the three most stable M06-2X/6-31G (d, p)/SDD optimized structures found for each mercury-peptide complex. All tetrapeptides are in a zwitterion protonation state and cysteine thiol and glutamic acid carboxylate groups are deprotonated.  Table 1 provides some thermodynamic values used to determine *G*_soln_ (1) for each complex in Figure 6. Coordinates and select Gaussian output information for all structures in Figure 6 are provided in the supplementary information (Table S1).

#### 3.6.1. 1 : 1 Hg(XCGC) Complexes

Stable 1 : 1 complexes (Figure 6) form with two S‒Hg bonds (∼2.40 Å) having an S‒Hg‒S angle just bent from linear (167°–178°). Another common structural feature among the complexes includes O---Hg interactions (∼2.7 Å) between the second peptide carbonyl oxygen and the dithiolated mercury. All these optimized structures show a *β*-turn that is also stabilized by intramolecular hydrogen bonds between the peptide bonds, as well as that between the *N*-terminal ammonium and *C*-terminal carboxylate groups. The above structural features are well exemplified in each complex particularly the Hg(**GCGC**) complexes which have no *N*-terminal amino acid side functionality (Figure 6(a)).

Table 1 provides some thermodynamic values and Gibbs energies in solution (*G*_soln_) for each complex as shown in Figure 6. Since relative energies were calculated and not complexation energies, thermodynamic comparisons are limited to optimized mercury complexes with the same peptide. From Gibbs energy in solution, Hg(**GCGC**)-1 is the most stable by 2.1 kJ/mol over Hg(**GCGC**)-2 and by 3.3 kJ/mol over Hg(**GCGC**)-3. This energy range is small. When considering the sum of electronic MP2 energy and zero-point vibrational energy (ZPVE) (Δ*H*_0_, change in enthalpy at 0 K using data in Table 1), Hg(**GCGC**)-2 is more stable than Hg(**GCGC**)-1 by 78 kJ/mol and Hg(**GCGC**)-3 is more stable than Hg(**GCGC**)-1 by 42 kJ/mol. Hg(**GCGC**)-1 has the most negative Gibbs energy of solvation indicating its stability derives mainly from favorable solvation. The intrinsic stability, via Δ*H*_0_, of Hg(**GCGC**)-2 may be due to both oxygen atoms of the *C*-terminal carboxylate being involved in intramolecular interactions (O---Hg and O---HN).

Figure 6(b) shows optimized structures for Hg(**ECGC**) complexes. With a glutamyl residue at the *N*-terminal, the deprotonated glutamate group shows an affinity for the dithiolated mercury and the ammonium group. This flexible side-chain carboxylate O-donor interacts with the coordinated mercury with O---Hg distances of 2.68 Å in Hg(**ECGC)**-1, and 2.54 Å in Hg(**ECGC**)-3. Alternatively, hydrogen bonds with the *N*-terminal ammonium group and enables the *C*-terminal carboxylate oxygen to interact with the thiolated mercury [Hg(**ECGC**)-2] instead. Although *O*-donors have a lower affinity for mercury(II) than *S*-donors, these optimized structures indicate that they can provide auxiliary binding to stabilize the thiolated mercury. From Gibbs energy in solution, Hg(**ECGC**)-1 is the most stable by 4.1 kJ/mol over Hg(**ECGC**)-2 and by 6.0 kJ/mol over Hg(**ECGC**)-3. From Δ*H*_0_, Hg(**ECGC**)-1 is the most stable by 43 kJ/mol over Hg(**ECGC**)-2 and by 27 kJ/mol over Hg(**ECGC**)-3. The intrinsic stability in Hg(**ECGC**) complexes is maximum when the glutamate carboxylate interacts with mercury. Hg(**ECGC**)-1 also has the lowest Gibbs energy of solvation indicating its stability derives mainly from strength of the glutamate carboxylate to mercury interaction.

The optimized structures for Hg(**HCGC**) complexes are shown in Figure 6(c). These structures show that the *N*-terminal histidyl side-chain group is also flexible and contributes to complex stability by reinforcing the peptide turn structure via an N_im_-H---O hydrogen bond with the *C*-terminal carboxylate group [Hg(**HCGC**)-1 and Hg(**HCGC**)-2]. This hydrogen bonding with the *C*-terminal carboxylate oxygen is more stabilizing than when the N_im_-H forms a hydrogen bond with the first peptide bond as shown in Hg(**HCGC**)-3. These results show that the *N*-terminal histidyl residue can contribute to stabilizing the peptide turn structure via intramolecular hydrogen bonding. From Gibbs energy in solution, Hg(**HCGC**)-1 is the most stable by 3.3 kJ/mol over Hg(**HCGC**)-2 and by 8.6 kJ/mol over Hg(**HCGC**)-3. From Δ*H*_0_, Hg(**HCGC**)-2 is the most stable by 21 kJ/mol over Hg(**HCGC**)-1 and by 30 kJ/mol over Hg(**HCGC**)-3. The stability of the complex is favored when the imidazole N-H hydrogen interacts with the *C*-terminal carboxylate group. A stability decrease is predicted when the imidazole N-H instead interacts with the *N*-terminal amide carbonyl oxygen (Hg(**HCGC**)-3).

Figure 6(d) shows the optimized structures for the peptide **WCGC**, which contains an indole-*π* donor group. In complex Hg(**WCGC**)-1 the hydrogen of the cysteinyl beta carbon is 2.80 Å from the indole benzo ring and in complex Hg(**WCGC**)-3 they are 2.67 Å apart. These C_*β*_-H and indole-*π* interactions enable the indole ring to shield one end of the coordination site while the dithiolated mercury is additionally stabilized by two O-donors, an amide carbonyl and the *C*-terminal carboxylate. To determine the source of stability gained from the observed C_*β*_-H and indole interaction, atomic charges were predicted by conducting a Natural Population Analysis (NPA) [41] using MP2 densities from the gas-phase single-point calculations for these mercury complexes. The cysteine C_*β*_-H bond directed toward the indole ring is predicted to be more polar than the other C_*β*_-H bond, with *C*_*β*_ charge of −0.49 and H charge of +2.2. This indicates that the observed C_*β*_-H and indole interaction is similar to the well-established polarized C-H···*π* interaction, a weak hydrogen-bond-like interaction, which is often documented in proteins [19, 20] and identified as metal ligand cation-*π* interactions in some metalloproteins [43, 44]. In the Hg(**WCGC**)-2 complex, the indole ring is close to mercury having a distance of 3.27 Å between mercury and the fused carbon next to the ring nitrogen. In this case, the indole ring could participate in stabilizing the complex via cation-*π* interaction [14, 15], as indicated by the spectroscopic studies described in Sections 3.2, 3.4, and 3.5. Therefore, it is conceivable the flexible side-chain indole group in Hg(**WCGC**) complex can serve as a hydrophobic shield for the thiolated mercury. From Gibbs energy in solution, Hg(**WCGC**)-1 is the most stable by 1.5 kJ/mol over Hg(**WCGC**)-2 and by 3.5 kJ/mol over Hg(**WCGC**)-3. Using Δ*H*_0_, Hg(**WCGC**)-2 is the most stable by 0.5 kJ/mol over Hg(**WCGC**)-1 and by 1 kJ/mol over Hg(**WCGC**)-3. This Gibbs energy range is small (3.5 kJ/mol) and each structure has nearly the same Gibbs energy of solvation (1.3 kJ/mol difference). Interaction of the indole ring with the *N*-terminal Cys-C_*β*_-H or with the mercury also has nearly indistinguishable intrinsic stabilities. Furthermore, electronic interactions involving the pi system of the indole influence the stability of the complex since they exist in each of the three low energy structures.

## 4. Conclusions

By combining spectroscopic and theoretical studies, we have gained some structural insights into the associations of mercury(II) with four tetrapeptides containing a Cys-Gly-Cys motif and an *O*-, *N*-, *H-*or aromatic *π* donor group. UV-vis spectroscopic and ESI-MS studies indicate that mercury(II) binds to the cysteinyl thiolates of these peptides to form predominantly 1 : 1 dithiolated mercury complexes. The CD spectral data show that these tetrapeptides do not adopt preorganized turn structures. However, mercury(II) binding readily induces reversed peptide backbone turn structures. UV-vis absorption and fluorescence quenching of tryptophan indicate that the tryptophan indole ring in **WCGC** can participate in mercury cation-*π* interaction. Optimized stable structures of 1 : 1 mercuriated tetrapeptides corroborate the above spectroscopic results and show that each mercury-peptide complex adopts a reverse-turn secondary structure that is stabilized by intramolecular hydrogen bonds. They also show that the side-chain donor group present in the *N*-terminal residue is flexible and can stabilize the coordinated mercury or its local environment via O---Hg, hydrogen bonding, C-H---*π*, or mercury cation-*π* interactions. Additionally, the indole ring may act as a hydrophobic shield for the coordinated mercury. This peptide model-based study shows that amino acid side chains containing one or more donor groups can serve as auxiliary binding group(s) to enhance the immobilization of mercury(II).

## Figures and Tables

**Table 1:** Thermodynamic values for the determination of *G*_soln_ and relative Gibbs energy in solution for each complex.

Complex name	*E*_e_ (MP2, gas) (a. u.)	ZPVE unscaled (a. u.)	*G* (298 K, gas)^a^ (a. u.)	Δ*G* (solv) (a. u.)	*G* (solution) (a. u.)	Relative Δ*G* (soln) (kJ/mol)
Hg(GCGC)-1	−1932.47170	0.29444	−1932.24520	−0.11076	−1932.35595	0.00
Hg(GCGC)-2	−1932.50036	0.29341	−1932.27386	−0.08131	−1932.35517	2.06
Hg(GCGC)-3	−1932.48771	0.29436	−1932.26064	−0.09405	−1932.35469	3.30
Hg(ECGC)-1	−2198.66172	0.35246	−2198.38601	−0.14368	−2198.52969	0.00
Hg(ECGC)-2	−2198.64601	0.35295	−2198.37097	−0.15716	−2198.52813	4.07
Hg(ECGC)-3	−2198.65214	0.35311	−2198.37455	−0.15284	−2198.52739	6.02
Hg(HCGC)-1	−2196.27425	0.37506	−2195.97738	−0.10102	−2196.07840	0.00
Hg(HCGC)-2	−2196.28329	0.37603	−2195.98458	−0.09257	−2196.07716	3.27
Hg(HCGC)-3	−2196.27131	0.37578	−2195.97497	−0.10014	−2196.07511	8.64
Hg(WCGC)-1	−2333.55156	0.43329	−2333.20130	−0.08360	−2333.28490	0.00
Hg(WCGC)-2	−2333.55112	0.43332	−2333.20024	−0.08409	−2333.28434	1.47
Hg(WCGC)-3	−2333.55147	0.43343	−2333.19987	−0.08371	−2333.28358	3.46

^a^Using scaled zero-point vibrational energy (ZPVE).

## Data Availability

The mass spectral and computational data used to support the findings of this study are included within the supplementary information file.

## Supplementary Materials

Supplementary Materials Figure S1: ESI-MS spectra of all tetrapeptides. Table S1: Cartesian coordinates (Å) for all structures from modeling studies are provided.
