# Supplementary material for: Spectroscopic and Theoretical Studies of Hg(II) Complexation with Some Dicysteinyl Tetrapeptides
Source: Bioinorg Chem Appl. 2021 Jul 23;2021:9911474. doi: 10.1155/2021/9911474 (PMC8328728; doi:10.1155/2021/9911474)
Supplement: Supplementary Materials — Figure S1: ESI-MS spectra of all tetrapeptides. Table S1: Cartesian coordinates (Å) for all structures from modeling studies are provided. [file 9911474.f1.docx]

**Supplementary Material**

**Spectroscopic and theoretical studies of Hg(II) complexation with some dicysteinyl tetrapeptides**

Elliot Springfield^1^, Alana Willis^1^, John Merle^1^, Johanna Mazlo^2^, and Maria Ngu-Schwemlein^1^*

^1^Chemistry Department, Winston Salem State University, Winston-Salem, North Carolina, 27110, USA.
^2^Department of Chemistry and Biochemistry, University of North Carolina at Greensboro, North Carolina, 27402, USA

*Communicating author

Maria Ngu-Schwemlein

601 M.L. King Jr. Drive

W.B. Atkinson Science Bldg.

Winston-Salem, NC 27110

Phone: 1-336-7502919

[Schwemleinmn@wssu.edu](mailto:Mngu@usouthal.edu)


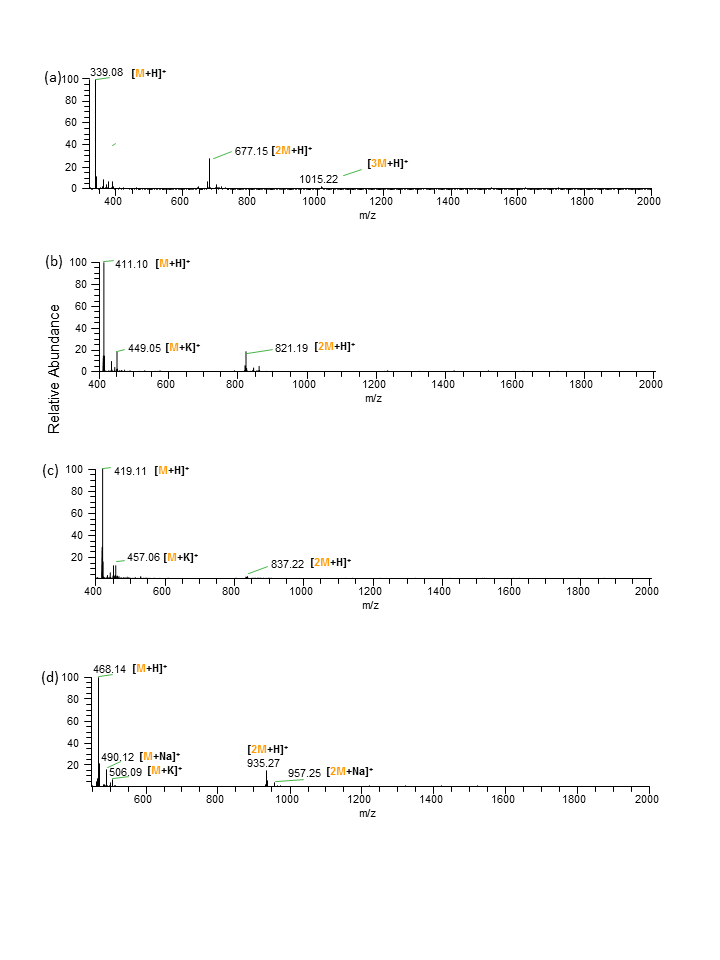


**Figure S1**: Electrospray ionization mass spectra of tetrapeptides, (a) **GCGC**, (b) **ECGC**, (c) **HCGC**, and (d) **WCGC**.

**Supplementary Information**

**Table 1.** Cartesian coordinates (Å) for all structures in Figure 6.

Hg(GCGC)-1

# gcgc_2minus_hg_csearch_1_169-b-f

__Requested operations__

Run with Gaussian 09revisionC.01.

`M062X/genecp freq scrf(smd,solvent=water,read) nosymmetry`

__Relevant magnitudes__

| Datum | Value |

|:-------------------------------------------------|--------------------------:|

| Charge | 0 |

| Multiplicity | 1 |

| Stoichiometry | C10H16HgN4O5S2 |

| Number of Basis Functions | 418 |

| Electronic Energy (Eh) | -1935.30587865 |

| Sum of electronic and zero-point Energies (Eh) | -1935.011441 |

| Sum of electronic and thermal Energies (Eh) | -1934.98881 |

| Sum of electronic and enthalpy Energies (Eh) | -1934.987866 |

| Sum of electronic and thermal Free Energies (Eh) | -1935.064654 |

| Number of Imaginary Frequencies | 0 |

| Mean of alpha and beta Electrons | 98 |

__Molecular Geometry in Cartesian Coordinates__

```xyz

C 3.015828 -2.090036 -1.620444

N 1.860311 -2.995819 -1.369262

H 0.969936 -2.438276 -1.421622

H 1.934537 -3.434957 -0.445615

H 2.841901 -1.585959 -2.570649

C 3.121775 -1.119476 -0.454216

O 3.729403 -1.435164 0.572712

N 2.480568 0.044085 -0.622034

H 1.910391 0.175206 -1.455841

C 2.400796 1.088973 0.388746

H 3.366952 1.132947 0.904243

C 1.333240 0.753212 1.447112

O 0.357808 1.485769 1.653211

H 2.333991 -0.960137 1.912891

N 1.506998 -0.401535 2.109312

C 0.466331 -0.917865 2.972233

H 0.860618 -1.776903 3.517300

C -0.805550 -1.369692 2.254965

O -1.798516 -1.658088 2.926751

C -1.842776 -1.819678 0.042251

N -0.748556 -1.443854 0.917097

H 0.125053 -1.247833 0.440891

C -3.160091 -1.083387 0.292299

H -2.068710 -2.887935 0.162973

H -3.865531 -1.451634 -0.452923

C -1.395998 -1.615035 -1.425054

O -2.236959 -1.830396 -2.308922

O -0.198276 -1.206085 -1.609366

H 3.924741 -2.686616 -1.666000

H 0.160641 -0.164432 3.702042

C 2.134124 2.443639 -0.268561

S 0.905571 2.456912 -1.637953

H 1.846755 3.157368 0.502912

H 3.059801 2.791185 -0.731853

S -3.156477 0.754856 0.205549

H -3.532467 -1.334313 1.284534

H 1.830474 -3.738842 -2.073335

Hg -1.049041 1.436060 -0.683334

```

__Frequencies__ (Top 10 out of 108)

```

1. 31.9854 cm-1 (Symmetry: A)

2. 47.2558 cm-1 (Symmetry: A)

3. 55.9657 cm-1 (Symmetry: A)

4. 61.2908 cm-1 (Symmetry: A)

5. 77.5435 cm-1 (Symmetry: A)

6. 79.3218 cm-1 (Symmetry: A)

7. 89.9345 cm-1 (Symmetry: A)

8. 101.8576 cm-1 (Symmetry: A)

9. 111.8289 cm-1 (Symmetry: A)

10. 122.0914 cm-1 (Symmetry: A)

```

***

Hg(GCGC)-2

# gcgc_hg_csearch_2_5-b-f

__Requested operations__

Run with Gaussian 09revisionC.01.

`M062X/genecp freq scrf(smd,solvent=water,read) nosymmetry`

__Relevant magnitudes__

| Datum | Value |

|:-------------------------------------------------|--------------------------:|

| Charge | 0 |

| Multiplicity | 1 |

| Stoichiometry | C10H16HgN4O5S2 |

| Number of Basis Functions | 418 |

| Electronic Energy (Eh) | -1935.30425572 |

| Sum of electronic and zero-point Energies (Eh) | -1935.010841 |

| Sum of electronic and thermal Energies (Eh) | -1934.988339 |

| Sum of electronic and enthalpy Energies (Eh) | -1934.987395 |

| Sum of electronic and thermal Free Energies (Eh) | -1935.063084 |

| Number of Imaginary Frequencies | 0 |

| Mean of alpha and beta Electrons | 98 |

__Molecular Geometry in Cartesian Coordinates__

```xyz

C 3.701361 0.035495 1.957183

N 2.382132 -0.221433 2.597674

H 2.020979 -1.142804 2.325379

H 1.641280 0.506371 2.324818

H 4.426097 -0.678746 2.341486

C 3.629789 -0.121399 0.443981

O 4.465823 -0.783573 -0.163238

N 2.618142 0.536929 -0.158683

H 1.912707 0.984731 0.427494

C 2.195027 0.189039 -1.507980

H 2.992605 0.451108 -2.214146

C 0.986482 1.078001 -1.827182

O -0.107072 0.606044 -2.163849

H 2.100093 2.705420 -1.344855

N 1.202323 2.397617 -1.700707

C 0.121145 3.376709 -1.717793

H 0.541482 4.357452 -1.932306

C -0.529483 3.397926 -0.335481

O -0.244108 4.244276 0.508689

C -1.736924 1.890434 1.197108

N -1.383227 2.372522 -0.125468

H -1.491317 1.711793 -0.889517

C -3.046790 1.111222 1.171792

H -1.874996 2.762272 1.847021

H -3.203781 0.665611 2.152873

C -0.570643 1.076610 1.824782

O 0.599235 1.542454 1.645455

O -0.835011 0.038559 2.465556

H 4.009360 1.050282 2.215875

H -0.595719 3.104154 -2.494583

C 1.896461 -1.305898 -1.704337

S 1.128893 -2.191902 -0.292486

H 1.291832 -1.424796 -2.603996

H 2.842571 -1.825771 -1.860919

S -3.199259 -0.189584 -0.125221

H -3.870651 1.798029 0.965485

H 2.486779 -0.213497 3.615959

Hg -1.026991 -1.186066 -0.160882

```

__Frequencies__ (Top 10 out of 108)

```

1. 39.1534 cm-1 (Symmetry: A)

2. 53.5706 cm-1 (Symmetry: A)

3. 68.5043 cm-1 (Symmetry: A)

4. 73.8485 cm-1 (Symmetry: A)

5. 82.5619 cm-1 (Symmetry: A)

6. 90.9426 cm-1 (Symmetry: A)

7. 92.4263 cm-1 (Symmetry: A)

8. 106.8119 cm-1 (Symmetry: A)

9. 122.2598 cm-1 (Symmetry: A)

10. 130.2922 cm-1 (Symmetry: A)

```

***

Hg(GCGC)-3

# gcgc_2minus_hg_csearch_1_22-b-f

__Requested operations__

Run with Gaussian 09revisionC.01.

`M062X/genecp freq scrf(smd,solvent=water,read) nosymmetry`

__Relevant magnitudes__

| Datum | Value |

|:-------------------------------------------------|--------------------------:|

| Charge | 0 |

| Multiplicity | 1 |

| Stoichiometry | C10H16HgN4O5S2 |

| Number of Basis Functions | 418 |

| Electronic Energy (Eh) | -1935.30422599 |

| Sum of electronic and zero-point Energies (Eh) | -1935.009868 |

| Sum of electronic and thermal Energies (Eh) | -1934.987261 |

| Sum of electronic and enthalpy Energies (Eh) | -1934.986317 |

| Sum of electronic and thermal Free Energies (Eh) | -1935.062436 |

| Number of Imaginary Frequencies | 0 |

| Mean of alpha and beta Electrons | 98 |

__Molecular Geometry in Cartesian Coordinates__

```xyz

C -3.287855 2.574231 -0.519358

N -2.243700 2.576079 -1.586492

H -2.420416 1.830699 -2.269662

H -1.269748 2.435474 -1.194270

H -4.228207 2.903325 -0.955227

C -3.488561 1.175038 0.049607

O -4.578995 0.617822 -0.008815

N -2.395007 0.617344 0.617929

H -1.508618 1.100619 0.478216

C -2.331496 -0.828498 0.798878

H -3.144049 -1.129962 1.471179

C -1.012221 -1.182759 1.497194

O -0.228344 -2.010335 1.019983

H -1.398836 0.147112 3.000276

N -0.742595 -0.538420 2.645753

C 0.549682 -0.691751 3.286702

H 0.497972 -0.244811 4.280945

C 1.721934 -0.047572 2.535937

O 2.875600 -0.384845 2.805934

C 2.211940 1.615169 0.709748

N 1.375319 0.888055 1.641916

H 0.395505 1.118948 1.565595

C 3.344309 0.811874 0.070463

H 2.694830 2.464301 1.211374

H 3.832233 1.480363 -0.639552

C 1.302145 2.222323 -0.387660

O 1.845763 2.891679 -1.275405

O 0.054034 1.958774 -0.301857

H -2.969932 3.271381 0.257359

H 0.792475 -1.749967 3.400193

C -2.508042 -1.612924 -0.509868

S -1.686829 -0.926888 -2.001513

H -2.192258 -2.643025 -0.345640

H -3.568925 -1.618072 -0.765289

S 2.921008 -0.753847 -0.795742

H 4.063485 0.527533 0.837935

H -2.265139 3.472954 -2.081273

Hg 0.593332 -0.698436 -1.300904

```

__Frequencies__ (Top 10 out of 108)

```

1. 41.3530 cm-1 (Symmetry: A)

2. 56.1100 cm-1 (Symmetry: A)

3. 61.9877 cm-1 (Symmetry: A)

4. 69.2639 cm-1 (Symmetry: A)

5. 76.7329 cm-1 (Symmetry: A)

6. 88.3814 cm-1 (Symmetry: A)

7. 95.7614 cm-1 (Symmetry: A)

8. 102.0784 cm-1 (Symmetry: A)

9. 115.6866 cm-1 (Symmetry: A)

10. 124.1068 cm-1 (Symmetry: A)

```

***

Hg(ECGC)-1

# ecgc_hg_csearch_3_61-b-f

__Requested operations__

Run with Gaussian 09revisionC.01.

`M062X/genecp freq scrf(smd,solvent=water,read) nosymmetry`

__Relevant magnitudes__

| Datum | Value |

|:-------------------------------------------------|--------------------------:|

| Charge | -1 |

| Multiplicity | 1 |

| Stoichiometry | C13H19HgN4O7S2(-1) |

| Number of Basis Functions | 503 |

| Electronic Energy (Eh) | -2201.94858284 |

| Sum of electronic and zero-point Energies (Eh) | -2201.596123 |

| Sum of electronic and thermal Energies (Eh) | -2201.568669 |

| Sum of electronic and enthalpy Energies (Eh) | -2201.567725 |

| Sum of electronic and thermal Free Energies (Eh) | -2201.655245 |

| Number of Imaginary Frequencies | 0 |

| Mean of alpha and beta Electrons | 117 |

__Molecular Geometry in Cartesian Coordinates__

```xyz

C 2.649946 0.999529 2.068518

N 1.683501 -0.102557 2.345929

H 0.666130 0.225578 2.417767

H 1.955250 -0.576839 3.210060

C 3.993360 0.425816 1.568626

C 2.074669 2.041107 1.107656

O 2.274189 3.236759 1.294758

N 1.400784 1.550506 0.040335

H 1.487874 0.546262 -0.104831

C 1.067548 2.376813 -1.107409

H 1.707191 3.265768 -1.077127

C -0.378203 2.874551 -1.109459

O -0.778326 3.604147 -2.019600

H -0.764726 1.831489 0.613095

N -1.149301 2.450991 -0.101581

C -2.586273 2.681154 -0.079515

H -2.836345 3.685786 0.267888

C -3.151913 1.668967 0.904187

O -3.613528 2.001414 1.991881

C -2.863412 -0.636277 1.562943

N -3.027341 0.370155 0.528481

H -2.568058 0.206593 -0.359716

C -2.732956 -2.045300 0.973208

H -3.758360 -0.630271 2.191224

H -3.013092 -2.758571 1.750468

C -1.655682 -0.333329 2.482245

O -1.542183 -1.035643 3.499169

O -0.851601 0.585754 2.112563

H 2.814649 1.521431 3.010541

H -2.996171 2.536419 -1.084380

C 1.366344 1.639589 -2.419006

S 0.139666 0.396176 -2.994939

H 2.360002 1.188178 -2.355497

H 1.380566 2.377665 -3.223219

S -1.050462 -2.616650 0.482188

H -3.430663 -2.165613 0.142297

H 4.760066 1.156035 1.839992

H 4.198684 -0.474174 2.158518

C 4.170472 0.133034 0.073094

H 4.057971 1.059309 -0.506684

H 5.204163 -0.183996 -0.087683

C 3.279253 -0.903559 -0.619511

O 3.650280 -1.330680 -1.724641

O 2.164520 -1.229796 -0.062359

H 1.751703 -0.783615 1.557588

Hg -0.262693 -1.120784 -1.194202

```

__Frequencies__ (Top 10 out of 132)

```

1. 29.9673 cm-1 (Symmetry: A)

2. 40.6601 cm-1 (Symmetry: A)

3. 45.2394 cm-1 (Symmetry: A)

4. 54.1709 cm-1 (Symmetry: A)

5. 57.7904 cm-1 (Symmetry: A)

6. 69.8581 cm-1 (Symmetry: A)

7. 73.8270 cm-1 (Symmetry: A)

8. 85.4935 cm-1 (Symmetry: A)

9. 90.1622 cm-1 (Symmetry: A)

10. 97.7876 cm-1 (Symmetry: A)

```

***

Hg(ECGC)-2

# ecgc_hg_csearch_3_194-b-f

__Requested operations__

Run with Gaussian 09revisionC.01.

`M062X/genecp freq scrf(smd,solvent=water,read) nosymmetry`

__Relevant magnitudes__

| Datum | Value |

|:-------------------------------------------------|--------------------------:|

| Charge | -1 |

| Multiplicity | 1 |

| Stoichiometry | C13H19HgN4O7S2(-1) |

| Number of Basis Functions | 503 |

| Electronic Energy (Eh) | -2201.95762067 |

| Sum of electronic and zero-point Energies (Eh) | -2201.604673 |

| Sum of electronic and thermal Energies (Eh) | -2201.577104 |

| Sum of electronic and enthalpy Energies (Eh) | -2201.57616 |

| Sum of electronic and thermal Free Energies (Eh) | -2201.664936 |

| Number of Imaginary Frequencies | 0 |

| Mean of alpha and beta Electrons | 117 |

__Molecular Geometry in Cartesian Coordinates__

```xyz

C -3.647143 -1.022432 -1.390474

N -5.122695 -1.182598 -1.282143

H -5.459923 -0.331010 -0.734502

H -5.356544 -2.036121 -0.767661

C -3.322726 0.398338 -1.877986

C -3.071134 -1.398925 -0.024081

O -3.783940 -1.933418 0.825537

N -1.768858 -1.121228 0.156185

H -1.222654 -0.708496 -0.601879

C -1.080835 -1.486921 1.381759

H -1.860538 -1.692824 2.123727

C -0.283019 -0.293871 1.924284

O 0.912490 -0.398775 2.234378

H -1.945708 0.937888 1.779325

N -0.953433 0.860931 2.040273

C -0.265644 2.074717 2.424264

H -1.017971 2.843408 2.615407

C 0.697313 2.627794 1.371707

O 1.475032 3.537675 1.672738

C 1.363624 2.375237 -1.029893

N 0.587007 2.084883 0.154500

H -0.099111 1.355369 0.006913

C 2.880066 2.400873 -0.830931

H 1.095294 3.361624 -1.430573

H 3.319273 2.585938 -1.812054

C 0.980276 1.328024 -2.107403

O 0.168139 0.413445 -1.759979

O 1.528227 1.446860 -3.218241

H -3.285594 -1.757908 -2.113436

H 0.312162 1.927050 3.339651

C -0.235658 -2.754530 1.247750

S 0.825000 -2.903452 -0.241833

H -0.923202 -3.601184 1.175359

H 0.359897 -2.877132 2.153177

S 3.684295 0.911704 -0.109607

H 3.144641 3.225581 -0.169429

H -2.375229 0.362383 -2.421266

H -4.088691 0.661732 -2.613526

C -3.213558 1.477924 -0.788610

H -2.188299 1.542482 -0.422001

H -3.444518 2.449947 -1.237711

C -4.142688 1.313321 0.416699

O -3.673942 1.552674 1.555940

O -5.345471 0.965570 0.197497

H -5.548858 -1.208312 -2.210776

Hg 2.108302 -0.882628 -0.218236

```

__Frequencies__ (Top 10 out of 132)

```

1. 28.3924 cm-1 (Symmetry: A)

2. 31.2023 cm-1 (Symmetry: A)

3. 38.9572 cm-1 (Symmetry: A)

4. 47.3202 cm-1 (Symmetry: A)

5. 59.0521 cm-1 (Symmetry: A)

6. 61.6814 cm-1 (Symmetry: A)

7. 68.4076 cm-1 (Symmetry: A)

8. 78.0778 cm-1 (Symmetry: A)

9. 87.4494 cm-1 (Symmetry: A)

10. 91.9740 cm-1 (Symmetry: A)

```

***

Hg(ECGC)-3

# ecgc_hg_csearch_3_302-b-f

__Requested operations__

Run with Gaussian 09revisionC.01.

`M062X/genecp freq scrf(smd,solvent=water,read) nosymmetry`

__Relevant magnitudes__

| Datum | Value |

|:-------------------------------------------------|--------------------------:|

| Charge | -1 |

| Multiplicity | 1 |

| Stoichiometry | C13H19HgN4O7S2(-1) |

| Number of Basis Functions | 503 |

| Electronic Energy (Eh) | -2201.95178917 |

| Sum of electronic and zero-point Energies (Eh) | -2201.598684 |

| Sum of electronic and thermal Energies (Eh) | -2201.57144 |

| Sum of electronic and enthalpy Energies (Eh) | -2201.570496 |

| Sum of electronic and thermal Free Energies (Eh) | -2201.656547 |

| Number of Imaginary Frequencies | 0 |

| Mean of alpha and beta Electrons | 117 |

__Molecular Geometry in Cartesian Coordinates__

```xyz

C 1.765636 1.630753 2.263584

N 0.795869 1.685882 3.394468

H 0.487859 2.652308 3.535384

H 1.215967 1.329729 4.255846

C 2.174910 0.179678 2.028304

C 1.057299 2.378514 1.125088

O 0.443405 3.413275 1.399798

N 1.110052 1.871749 -0.117325

H 1.725888 1.069521 -0.342776

C 0.517469 2.606452 -1.226636

H 0.875158 3.642311 -1.195808

C -1.012044 2.695363 -1.193018

O -1.590156 3.413218 -2.013164

H -1.146759 1.423457 0.420944

N -1.654743 1.933786 -0.301124

C -3.106136 1.833423 -0.268140

H -3.573621 2.744791 0.111764

C -3.428257 0.689250 0.680491

O -4.065918 0.851644 1.715266

C -2.478116 -1.421004 1.381443

N -2.894674 -0.511160 0.327671

H -2.340036 -0.507224 -0.520459

C -1.872032 -2.707442 0.814553

H -3.362599 -1.707498 1.957600

H -1.905806 -3.465066 1.599478

C -1.497831 -0.749629 2.375918

O -1.048870 0.403474 2.064963

O -1.217631 -1.387202 3.402502

H 2.635478 2.232879 2.543662

H -3.486688 1.630734 -1.275319

C 0.951188 2.003678 -2.571158

S 0.025084 0.536629 -3.180700

H 2.021041 1.787837 -2.537388

H 0.784820 2.760177 -3.339976

S -0.090807 -2.670999 0.344775

H -2.477743 -3.063373 -0.021101

H 2.302435 -0.283802 3.010588

H 1.345102 -0.351335 1.548172

C 3.488914 0.022331 1.228065

H 3.835720 0.996465 0.866420

H 4.269338 -0.399301 1.861818

C 3.267870 -0.876871 0.016493

O 2.561393 -0.356510 -0.923439

O 3.732026 -2.028325 -0.005168

H -0.047523 1.090511 3.087968

Hg 0.153885 -1.030001 -1.371856

```

__Frequencies__ (Top 10 out of 132)

```

1. 43.0723 cm-1 (Symmetry: A)

2. 45.4360 cm-1 (Symmetry: A)

3. 57.8613 cm-1 (Symmetry: A)

4. 61.3958 cm-1 (Symmetry: A)

5. 66.8653 cm-1 (Symmetry: A)

6. 77.0451 cm-1 (Symmetry: A)

7. 82.5897 cm-1 (Symmetry: A)

8. 82.8542 cm-1 (Symmetry: A)

9. 95.6385 cm-1 (Symmetry: A)

10. 103.6410 cm-1 (Symmetry: A)

```

***

Hg(HCGC)-1

# hcgc2_hg_csearch_10_71-b-f

__Requested operations__

Run with Gaussian 09revisionC.01.

`M062X/genecp freq scrf(smd,solvent=water,read) nosymmetry`

__Relevant magnitudes__

| Datum | Value |

|:-------------------------------------------------|--------------------------:|

| Charge | 0 |

| Multiplicity | 1 |

| Stoichiometry | C14H20HgN6O5S2 |

| Number of Basis Functions | 522 |

| Electronic Energy (Eh) | -2199.56129532 |

| Sum of electronic and zero-point Energies (Eh) | -2199.186234 |

| Sum of electronic and thermal Energies (Eh) | -2199.15874 |

| Sum of electronic and enthalpy Energies (Eh) | -2199.157795 |

| Sum of electronic and thermal Free Energies (Eh) | -2199.245673 |

| Number of Imaginary Frequencies | 0 |

| Mean of alpha and beta Electrons | 119 |

__Molecular Geometry in Cartesian Coordinates__

```xyz

N -0.144518 -1.476852 1.665437

C 1.209806 -1.583034 2.202150

H 1.133460 -1.771002 3.279140

C 1.992922 -0.270775 2.009759

O 2.936083 -0.184282 1.210751

H 0.763458 0.684035 3.316610

N 1.591784 0.782658 2.734351

C 2.107117 2.095461 2.404095

H 1.720982 2.813637 3.129975

C 1.708869 2.583473 1.010682

O 2.357141 3.474417 0.464172

C -0.101556 2.537651 -0.657718

N 0.607321 2.007537 0.485854

C 0.083113 1.729074 -1.956412

H 0.231439 3.566130 -0.830580

H -0.287813 2.335303 -2.784478

C -1.615201 2.526595 -0.336512

O -1.952541 2.150862 0.820112

O -2.392249 2.827390 -1.272208

H 3.196795 2.106994 2.455458

C 1.944852 -2.756460 1.552192

S 1.710812 -2.980182 -0.260407

H 1.572406 -3.685870 1.987627

H 3.006380 -2.670021 1.781640

S 1.812907 1.277974 -2.381049

H -0.529404 0.823674 -1.928820

N -3.305810 0.234785 2.130384

C -2.522318 -0.922326 1.619681

C -1.149120 -0.848105 2.290297

O -1.021731 -0.253449 3.364628

C -2.500909 -0.918754 0.082021

C -3.850139 -0.627447 -0.495190

N -4.200627 0.594977 -1.022529

C -4.989452 -1.388477 -0.570473

C -5.499017 0.512748 -1.391997

N -6.017946 -0.672478 -1.137209

H -2.998220 1.104552 1.627366

H -3.015784 -1.828294 1.983356

H -1.773232 -0.181883 -0.271166

H -2.170672 -1.905322 -0.255634

H -5.113312 -2.414106 -0.251223

H -3.122680 0.354066 3.133009

H -4.307084 0.080835 1.982125

H -0.307542 -1.929710 0.770469

H 0.007443 1.504753 1.131092

Hg 1.924987 -0.778109 -1.190670

H -3.599327 1.424516 -1.116414

H -6.016575 1.343889 -1.848898

```

__Frequencies__ (Top 10 out of 138)

```

1. 21.0102 cm-1 (Symmetry: A)

2. 34.4253 cm-1 (Symmetry: A)

3. 44.8600 cm-1 (Symmetry: A)

4. 56.9135 cm-1 (Symmetry: A)

5. 64.1691 cm-1 (Symmetry: A)

6. 66.3554 cm-1 (Symmetry: A)

7. 76.7408 cm-1 (Symmetry: A)

8. 85.5422 cm-1 (Symmetry: A)

9. 100.1926 cm-1 (Symmetry: A)

10. 103.0253 cm-1 (Symmetry: A)

```

***

Hg(HCGC)-2

# hcgc2_hg_csearch_10_42-b-f

__Requested operations__

Run with Gaussian 09revisionC.01.

`M062X/genecp freq scrf(smd,solvent=water,read) nosymmetry`

__Relevant magnitudes__

| Datum | Value |

|:-------------------------------------------------|--------------------------:|

| Charge | 0 |

| Multiplicity | 1 |

| Stoichiometry | C14H20HgN6O5S2 |

| Number of Basis Functions | 522 |

| Electronic Energy (Eh) | -2199.56201808 |

| Sum of electronic and zero-point Energies (Eh) | -2199.185987 |

| Sum of electronic and thermal Energies (Eh) | -2199.158707 |

| Sum of electronic and enthalpy Energies (Eh) | -2199.157762 |

| Sum of electronic and thermal Free Energies (Eh) | -2199.244514 |

| Number of Imaginary Frequencies | 0 |

| Mean of alpha and beta Electrons | 119 |

__Molecular Geometry in Cartesian Coordinates__

```xyz

N 0.736635 2.288116 -0.057242

C -0.443414 2.789271 -0.748487

H -0.430495 3.885775 -0.720664

C -1.650681 2.315947 0.073657

O -2.530460 1.594564 -0.415285

H -0.871031 3.233051 1.705603

N -1.671315 2.721185 1.352614

C -2.604177 2.172769 2.331224

H -2.683306 2.865232 3.167206

C -2.057372 0.839555 2.838815

O -1.445804 0.750981 3.901114

C -1.435509 -1.386050 2.004682

N -2.266012 -0.197225 1.996974

C -2.129111 -2.561023 1.328200

H -1.257771 -1.663558 3.050953

H -2.973307 -2.882612 1.942338

C -0.021065 -1.095328 1.423859

O 0.574430 -2.020747 0.826979

O 0.436457 0.063802 1.639487

H -3.581792 2.050358 1.861186

C -0.548234 2.374974 -2.223456

S 0.051377 0.700389 -2.669656

H 0.087156 3.039736 -2.810432

H -1.578838 2.515913 -2.551067

S -2.872024 -2.230978 -0.324553

H -1.420684 -3.384024 1.250055

N 2.845627 1.413662 1.562756

C 3.083135 1.667970 0.107982

C 1.982366 2.529086 -0.511498

O 2.254280 3.342312 -1.389217

C 3.166159 0.320562 -0.633640

C 4.011845 -0.674787 0.089799

N 3.447343 -1.715668 0.789538

C 5.361038 -0.779480 0.317457

C 4.455117 -2.393196 1.384293

N 5.633345 -1.860459 1.124099

H 2.683462 2.286526 2.075644

H 4.021879 2.215786 0.027762

H 2.154038 -0.083922 -0.743491

H 3.549878 0.523488 -1.636444

H 6.143121 -0.137891 -0.063901

H 3.678940 0.965551 1.961143

H 2.028656 0.769521 1.708740

H 0.589089 1.438964 0.496108

H -2.747037 0.014078 1.127758

Hg -1.353808 -0.754449 -1.422626

H 2.437984 -1.906542 0.841074

H 4.277629 -3.268496 1.992351

```

__Frequencies__ (Top 10 out of 138)

```

1. 31.3153 cm-1 (Symmetry: A)

2. 41.4088 cm-1 (Symmetry: A)

3. 54.2957 cm-1 (Symmetry: A)

4. 55.7108 cm-1 (Symmetry: A)

5. 73.6368 cm-1 (Symmetry: A)

6. 76.3583 cm-1 (Symmetry: A)

7. 81.4238 cm-1 (Symmetry: A)

8. 90.4868 cm-1 (Symmetry: A)

9. 96.7127 cm-1 (Symmetry: A)

10. 100.7203 cm-1 (Symmetry: A)

```

***

Hg(HCGC)-3

# hcgc2_2minus_hg_csearch_1_15-b-f

__Requested operations__

Run with Gaussian 09revisionC.01.

`M062X/genecp freq scrf(smd,solvent=water,read) nosymmetry`

__Relevant magnitudes__

| Datum | Value |

|:-------------------------------------------------|--------------------------:|

| Charge | 0 |

| Multiplicity | 1 |

| Stoichiometry | C14H20HgN6O5S2 |

| Number of Basis Functions | 522 |

| Electronic Energy (Eh) | -2199.56310877 |

| Sum of electronic and zero-point Energies (Eh) | -2199.187331 |

| Sum of electronic and thermal Energies (Eh) | -2199.159708 |

| Sum of electronic and enthalpy Energies (Eh) | -2199.158763 |

| Sum of electronic and thermal Free Energies (Eh) | -2199.247974 |

| Number of Imaginary Frequencies | 0 |

| Mean of alpha and beta Electrons | 119 |

__Molecular Geometry in Cartesian Coordinates__

```xyz

N -0.985679 -0.010379 1.611882

C -0.468303 -1.370039 1.739256

H -0.822203 -1.781910 2.691319

C 1.062153 -1.323199 1.809634

O 1.766312 -1.985678 1.040930

H 0.987217 0.038825 3.331979

N 1.597466 -0.515059 2.741808

C 3.028091 -0.276542 2.767859

H 3.283817 0.216803 3.707041

C 3.554964 0.592728 1.621484

O 4.766279 0.647229 1.403602

C 2.780214 2.125365 -0.209657

N 2.617289 1.266908 0.943411

C 3.713987 1.593549 -1.298777

H 3.192443 3.097754 0.091155

H 4.731796 1.551466 -0.911663

C 1.377460 2.408610 -0.798732

O 0.397206 1.804922 -0.238934

O 1.306328 3.167596 -1.771675

H 3.567809 -1.224135 2.721269

C -0.959055 -2.302281 0.622059

S -1.039949 -1.587885 -1.065877

H -1.989155 -2.589696 0.842486

H -0.347372 -3.204264 0.621575

S 3.388350 -0.080699 -1.987248

H 3.679409 2.315097 -2.115872

N -2.287603 1.691530 -0.348563

C -2.836264 1.497655 1.031451

C -2.321987 0.181849 1.636312

O -3.097323 -0.629739 2.134732

C -4.365612 1.530729 0.973871

C -4.939715 0.619710 -0.062954

N -4.995391 -0.751242 0.041372

C -5.475136 0.897085 -1.295042

C -5.547000 -1.226174 -1.099433

N -5.856729 -0.258224 -1.937820

H -1.241683 1.865960 -0.379338

H -2.462411 2.327865 1.636554

H -4.746429 1.296780 1.970564

H -4.669932 2.552357 0.730741

H -5.611879 1.873221 -1.739980

H -2.753747 2.497295 -0.781554

H -2.488941 0.871966 -0.935747

H -0.423279 0.648832 1.075238

H 1.663160 1.200922 1.266645

Hg 1.165040 -0.701005 -1.397514

H -4.632639 -1.284595 0.823114

H -5.701882 -2.282536 -1.265195

```

__Frequencies__ (Top 10 out of 138)

```

1. 13.7194 cm-1 (Symmetry: A)

2. 33.9821 cm-1 (Symmetry: A)

3. 41.9665 cm-1 (Symmetry: A)

4. 54.8454 cm-1 (Symmetry: A)

5. 57.4809 cm-1 (Symmetry: A)

6. 62.6168 cm-1 (Symmetry: A)

7. 76.7553 cm-1 (Symmetry: A)

8. 77.6608 cm-1 (Symmetry: A)

9. 80.3896 cm-1 (Symmetry: A)

10. 83.3614 cm-1 (Symmetry: A)

```

***

Hg(WCGC)-1

# wcgc_hg_csearch_4_3-b-f

__Requested operations__

Run with Gaussian 09revisionC.01.

`M062X/genecp freq scrf(smd,solvent=water,read) nosymmetry`

__Relevant magnitudes__

| Datum | Value |

|:-------------------------------------------------|--------------------------:|

| Charge | 0 |

| Multiplicity | 1 |

| Stoichiometry | C19H23HgN5O5S2 |

| Number of Basis Functions | 593 |

| Electronic Energy (Eh) | -2337.10650913 |

| Sum of electronic and zero-point Energies (Eh) | -2336.67322 |

| Sum of electronic and thermal Energies (Eh) | -2336.643263 |

| Sum of electronic and enthalpy Energies (Eh) | -2336.642319 |

| Sum of electronic and thermal Free Energies (Eh) | -2336.734576 |

| Number of Imaginary Frequencies | 0 |

| Mean of alpha and beta Electrons | 132 |

__Molecular Geometry in Cartesian Coordinates__

```xyz

N -1.430954 0.465144 0.608642

C -1.013916 -0.615301 1.483442

H -1.628518 -0.586050 2.391663

C 0.452504 -0.391013 1.884888

O 1.370674 -1.117551 1.480850

H -0.126546 1.279895 2.866939

N 0.664344 0.663001 2.689409

C 2.002593 1.220964 2.832228

H 2.010945 1.896959 3.686190

C 2.300633 2.011659 1.565404

O 1.794909 3.120515 1.375777

C 3.144605 1.923759 -0.695151

N 3.072858 1.396328 0.650077

H 3.297550 0.416686 0.795330

C 4.004643 1.036926 -1.600426

H 3.619554 2.909590 -0.673156

H 5.052937 1.150543 -1.314178

C 1.758441 2.150200 -1.330502

O 0.798719 1.397870 -0.931201

O 1.665235 3.015974 -2.209961

H 2.719134 0.416130 2.996356

C -1.233508 -1.960809 0.785696

S -0.882699 -2.019649 -1.022685

H -2.294551 -2.216635 0.861830

H -0.656233 -2.725465 1.305651

S 3.719143 -0.780173 -1.547033

H 3.894675 1.399103 -2.624058

N -1.586812 2.365607 -1.385510

C -2.261294 2.640576 -0.085027

C -1.803867 1.682952 1.006340

O -1.853037 2.040008 2.189602

C -3.794445 2.564115 -0.247937

C -4.264634 1.201879 -0.662892

C -4.492860 0.766723 -1.946193

C -4.494196 0.063835 0.192559

N -4.844071 -0.559479 -1.940541

C -4.847820 -1.019820 -0.645994

C -4.424058 -0.141132 1.582127

C -5.130567 -2.295263 -0.141055

C -4.706354 -1.399285 2.087171

C -5.055738 -2.466920 1.231271

H -2.110987 1.658299 -1.919145

H -1.965295 3.640728 0.231181

H -4.233509 2.853349 0.711902

H -4.086472 3.316611 -0.986720

H -4.448194 1.318817 -2.875668

H -5.069420 -1.107223 -2.758976

H -4.138959 0.672550 2.244725

H -5.397620 -3.112317 -0.803489

H -4.656459 -1.573079 3.157402

H -5.268215 -3.441886 1.658372

H -1.433956 0.222650 -0.378422

H -0.556332 2.007411 -1.262210

H -1.570141 3.217818 -1.952842

Hg 1.366995 -1.151966 -1.192213

```

__Frequencies__ (Top 10 out of 159)

```

1. 29.6528 cm-1 (Symmetry: A)

2. 30.9682 cm-1 (Symmetry: A)

3. 49.3686 cm-1 (Symmetry: A)

4. 52.6772 cm-1 (Symmetry: A)

5. 56.9367 cm-1 (Symmetry: A)

6. 62.6895 cm-1 (Symmetry: A)

7. 69.9425 cm-1 (Symmetry: A)

8. 78.6122 cm-1 (Symmetry: A)

9. 84.9787 cm-1 (Symmetry: A)

10. 87.5005 cm-1 (Symmetry: A)

```

***

Hg(WCGC)-2

# wcgc_hg_csearch_4_142-b-f

__Requested operations__

Run with Gaussian 09revisionC.01.

`M062X/genecp freq scrf(smd,solvent=water,read) nosymmetry`

__Relevant magnitudes__

| Datum | Value |

|:-------------------------------------------------|--------------------------:|

| Charge | 0 |

| Multiplicity | 1 |

| Stoichiometry | C19H23HgN5O5S2 |

| Number of Basis Functions | 593 |

| Electronic Energy (Eh) | -2337.09737109 |

| Sum of electronic and zero-point Energies (Eh) | -2336.66405 |

| Sum of electronic and thermal Energies (Eh) | -2336.634096 |

| Sum of electronic and enthalpy Energies (Eh) | -2336.633151 |

| Sum of electronic and thermal Free Energies (Eh) | -2336.724832 |

| Number of Imaginary Frequencies | 0 |

| Mean of alpha and beta Electrons | 132 |

__Molecular Geometry in Cartesian Coordinates__

```xyz

N 0.308104 0.395536 1.829791

C 1.074755 -0.806338 2.208673

H 1.137726 -0.828815 3.303846

C 2.500505 -0.534372 1.713447

O 3.088299 0.456524 2.158012

H 2.398967 -2.014914 0.335071

N 3.014393 -1.317844 0.750366

C 4.105794 -0.857969 -0.095485

H 4.859613 -0.360174 0.519995

C 3.634763 0.124651 -1.177573

O 4.031008 0.045899 -2.338336

C 2.369980 2.236377 -1.495102

N 2.769953 1.066268 -0.737858

H 2.735821 1.228482 0.264193

C 1.402406 1.944116 -2.645202

H 3.255768 2.709111 -1.940762

H 1.796533 1.138941 -3.266746

C 1.729014 3.238254 -0.514040

O 1.318063 4.310078 -0.976142

O 1.644200 2.865985 0.709211

H 4.563874 -1.712283 -0.590843

C 0.379971 -2.112892 1.809802

S 0.210843 -2.582257 0.037189

H -0.619408 -2.092083 2.247279

H 0.930708 -2.935439 2.274257

S -0.341737 1.590364 -2.177763

H 1.336291 2.845822 -3.257733

N -0.925554 2.891165 1.217457

C -1.508228 2.050336 2.298594

C -0.799614 0.718142 2.548831

O -1.268809 -0.022435 3.405464

C -2.998290 1.816061 1.985578

C -3.213064 1.132698 0.667966

C -3.378932 1.749616 -0.549169

C -3.248558 -0.286125 0.407786

N -3.518872 0.812373 -1.539871

C -3.432764 -0.443423 -0.990159

C -3.199252 -1.434228 1.221645

C -3.539151 -1.703803 -1.595918

C -3.315952 -2.677958 0.627678

C -3.478201 -2.811331 -0.767968

H -1.377686 3.811760 1.242658

H -1.411924 2.617408 3.227504

H -3.416369 1.228568 2.806190

H -3.491352 2.792987 1.991674

H -3.421691 2.805661 -0.782676

H -3.611473 1.017814 -2.525104

H -3.075586 -1.338131 2.296155

H -3.683735 -1.797524 -2.667700

H -3.279334 -3.571421 1.242964

H -3.563026 -3.803399 -1.200181

H 0.831646 1.161748 1.397216

H -1.132688 2.486260 0.289907

H 0.130363 3.038890 1.234847

Hg -0.160874 -0.474465 -1.010579

```

__Frequencies__ (Top 10 out of 159)

```

1. 21.9877 cm-1 (Symmetry: A)

2. 47.1321 cm-1 (Symmetry: A)

3. 50.4310 cm-1 (Symmetry: A)

4. 56.3914 cm-1 (Symmetry: A)

5. 58.9228 cm-1 (Symmetry: A)

6. 62.9667 cm-1 (Symmetry: A)

7. 74.3809 cm-1 (Symmetry: A)

8. 83.2147 cm-1 (Symmetry: A)

9. 87.4663 cm-1 (Symmetry: A)

10. 95.2727 cm-1 (Symmetry: A)

```

***

Hg(WCGC)-3

# wcgc_2minus_hg_csearch_1_174-b-f

__Requested operations__

Run with Gaussian 09revisionC.01.

`M062X/genecp freq scrf(smd,solvent=water,read) nosymmetry`

__Relevant magnitudes__

| Datum | Value |

|:-------------------------------------------------|--------------------------:|

| Charge | 0 |

| Multiplicity | 1 |

| Stoichiometry | C19H23HgN5O5S2 |

| Number of Basis Functions | 593 |

| Electronic Energy (Eh) | -2337.10634466 |

| Sum of electronic and zero-point Energies (Eh) | -2336.672911 |

| Sum of electronic and thermal Energies (Eh) | -2336.643232 |

| Sum of electronic and enthalpy Energies (Eh) | -2336.642287 |

| Sum of electronic and thermal Free Energies (Eh) | -2336.733072 |

| Number of Imaginary Frequencies | 0 |

| Mean of alpha and beta Electrons | 132 |

__Molecular Geometry in Cartesian Coordinates__

```xyz

N -0.746963 0.727632 0.343659

C -0.564344 -0.080285 1.541254

H -1.152658 0.382563 2.342801

C 0.911823 -0.029967 1.962040

O 1.646479 -1.027867 1.916242

H 0.693495 1.956220 2.291431

N 1.345061 1.176904 2.357373

C 2.762757 1.469939 2.536311

H 2.866617 2.348161 3.170487

C 3.368137 1.766022 1.166518

O 3.591847 2.911934 0.785383

C 3.594932 0.684946 -1.045285

N 3.559544 0.659214 0.411374

H 3.302724 -0.227566 0.836334

C 4.272260 -0.561364 -1.617404

H 4.177721 1.559434 -1.348811

H 5.354017 -0.448668 -1.513932

C 2.158023 0.910511 -1.573747

O 1.691480 2.078206 -1.407183

O 1.526242 -0.032925 -2.105608

H 3.247794 0.614167 3.009820

C -1.082572 -1.508084 1.343509

S -0.838246 -2.286044 -0.303512

H -2.168507 -1.496238 1.466325

H -0.659524 -2.140194 2.124973

S 3.924098 -2.182551 -0.813455

H 4.039924 -0.623457 -2.681177

N -0.792425 2.162022 -2.076164

C -1.472613 2.728832 -0.885319

C -1.054882 2.032677 0.402290

O -1.072907 2.670510 1.460142

C -3.004649 2.684694 -1.043401

C -3.555427 1.292406 -1.081379

C -3.754849 0.506203 -2.189229

C -3.935109 0.491062 0.054304

N -4.240020 -0.722095 -1.814965

C -4.353678 -0.765232 -0.446829

C -3.961612 0.719730 1.441534

C -4.797968 -1.793283 0.393793

C -4.405542 -0.292728 2.276783

C -4.819746 -1.538008 1.755599

H -0.961928 2.772884 -2.880392

H -1.141328 3.764062 -0.796616

H -3.430353 3.241703 -0.202898

H -3.254021 3.230024 -1.959242

H -3.605058 0.741108 -3.234621

H -4.456380 -1.480922 -2.445662

H -3.632885 1.672854 1.848557

H -5.117118 -2.748357 -0.011105

H -4.433840 -0.131554 3.349692

H -5.161603 -2.311204 2.436224

H -0.794928 0.208932 -0.530064

H -1.165567 1.238658 -2.326022

H 0.303067 2.097121 -1.875784

Hg 1.538219 -2.186380 -0.601849

```

__Frequencies__ (Top 10 out of 159)

```

1. 32.2474 cm-1 (Symmetry: A)

2. 38.3566 cm-1 (Symmetry: A)

3. 56.4619 cm-1 (Symmetry: A)

4. 60.4659 cm-1 (Symmetry: A)

5. 66.4707 cm-1 (Symmetry: A)

6. 73.2907 cm-1 (Symmetry: A)

7. 81.1944 cm-1 (Symmetry: A)

8. 83.1448 cm-1 (Symmetry: A)

9. 89.3003 cm-1 (Symmetry: A)

10. 105.1262 cm-1 (Symmetry: A)

```

***

Structure information generated using ESIGEN (<https://esigen.herokuapp.com/>) J. Chem. Inf. Model., **2018**, 58 (3), pp 561–564. DOI: [10.1021/acs.jcim.7b00714](https://pubs.acs.org/doi/10.1021/acs.jcim.7b00714).
